# Bystander Effects and Unintended Consequences: Time to Include the Spleen in Radiation Therapy Planning

**DOI:** 10.3389/fonc.2020.01171

**Published:** 2020-07-16

**Authors:** Matthew S. Katz

**Affiliations:** Department of Radiation Medicine, Lowell General Hospital, Lowell, MA, United States

**Keywords:** spleen, radiation therapy, lymphopenia, esophageal cancer, lung cancer, pancreatic cancer, gastric cancer, vaccination

During residency through 2003, I learned about the historical role of radiation treating the spleen on the lymphoma service, including use of low-dose radiation as palliative treatment with potential significant toxicity (1). But when treating gastrointestinal malignancies, there was no research at the time indicating concern about the potential effects of splenic irradiation. As radiation therapy has evolved over the past two decades, our ability to accurately shape high dose radiation fields has improved substantially.

In the era of intensity modulated radiation therapy (IMRT) and stereotactic body radiotherapy (SBRT), we have mostly ignored the spleen until recently. The time has come to contour it, so that treatment planning systems include splenic dose in plan optimization because of its potential to help or harm our patients.

## Spleen as Innocent Bystander

As IMRT use increased, the question became meaningful enough that I reviewed a small number of patients receiving abdominal radiation at my hospital 2007-8 and found a Dmean of 19 Gy and V20Gy of 82% for distal esophageal cancer patients (2). We didn't have enough patients to look at clinical outcomes but that seemed like a meaningful dose particularly for immune cells and spleen function. Reviewing the literature, I learned that Drs. C. Norman Coleman and Henry Kaplan identified splenic atrophy with radiation therapy in 1980 (3), subsequently demonstrating organ injury and a risk of sepsis (4, 5). There are well-documented risks of sepsis after splenectomy (6), and I didn't want the radiation to contribute to that risk. Since 2011, I have recommended pneumococcal vaccinations for my patients with upper abdominal malignancies before receiving chemoradiation on first principles, initially in the absence of any good data. Recent research confirms an association with higher splenic doses and risk of infection in pediatric cancer survivors that only increases with time, suggesting immunization has value (7).

Over the past decade, QUANTEC provided no guidance at all (8). RTOG consensus contouring guidelines show it in the figures but don't mention any detail (9). If the physician or dosimetrist doesn't contour and place constraints on the spleen, treatment planning system software will consider the spleen a safe part of the body to push dose to avoid other important intra-abdominal organs.

Now, there are increasing data emerging suggesting that we should care about potential negative effects of splenic radiation with solid malignancies. In gastric cancer, a small study showed chemoradiation had a splenic D_mean_ of 40 Gy with mean 37% volume loss and a high risk of pneumonia (10). For esophageal cancer chemoradiation, higher spleen dose is associated with a higher risk of lymphopenia in one study and lower toxicity in another (11, 12). Distal esophageal cancers and larger irradiated volumes are more likely to have severe lymphopenia, suggesting lower dose to normal tissue may matter (13, 14). Lymphopenia is also associated with lower survival (13). Similar data are emerging in pancreatic cancer chemoradiation (15–18), with lower rates of lymphopenia using stereotactic body techniques (19, 20). Splenic dose may also be clinically relevant in hepatoma (21).

Lung cancer research has mostly ignored potential splenic radiation toxicity. One study suggests decreased spleen volume with chemoradiation for non-small cell lung cancer but did not look at spleen dose or location of the primary tumor (22). Some interest in bone marrow toxicity has focused upon the vertebral bodies without evaluating splenic dose (23, 24). Cardiac dose and lung also has been associated with hematologic toxicity but did not assess the spleen or primary tumor location (25, 26). It is possible that the association of lymphopenia with higher cardiac dose occurs in left lower lobe primary tumors, where the spleen may also receive higher doses. Without including the spleen in treatment planning, any potential detrimental effects for lung cancer patients will remain unknown.

## A Role in Cancer Control?

The spleen also may play an important role in effective cancer treatment, especially in the immunotherapy era (27). Treatment-related lymphopenia is associated with worse disease progression and survival in esophageal, pancreatic, and lung cancer (13, 16, 28–30). The spleen is an important source of tumor-associated macrophages and neutrophils (31), which may contribute to tumor progression and death via decreased immune surveillance or pro-inflammatory stimulation for tumor growth (32–37). Radiation can also induce regulatory T cells (38), which may alter peripheral blood immunophenotype in a tissue-specific fashion (39).

Based upon the currently published literature, I can't predict what low doses of splenic radiation may do to worsen or improve cancer control. But it seems the spleen deserves more attention if it could help lessen treatment toxicity and may factor into efficacy.

Our treatment planning systems will not consider the spleen of any clinical value unless we do. The time has come to seriously evaluate splenic dose in solid malignancies. We have ample opportunity for more retrospective and prospective studies so we can learn how to better address the immunologic health of our patients receiving radiation therapy.

## Author Contributions

The author confirms being the sole contributor of this work and has approved it for publication.

## Conflict of Interest

The author declares that the research was conducted in the absence of any commercial or financial relationships that could be construed as a potential conflict of interest.
